# Development of Covalent Triazine Frameworks as Heterogeneous Catalytic Supports

**DOI:** 10.3390/polym11081326

**Published:** 2019-08-09

**Authors:** Norini Tahir, Chidharth Krishnaraj, Karen Leus, Pascal Van Der Voort

**Affiliations:** Center for Ordered Materials, Organometallics and Catalysis (COMOC), Ghent University, Krijgslaan 281 (S3), 9000 Ghent, Belgium

**Keywords:** covalent triazine frameworks, heterogeneous catalysis, catalytic supports, metal catalysis, organic synthesis

## Abstract

Covalent triazine frameworks (CTFs) are established as an emerging class of porous organic polymers with remarkable features such as large surface area and permanent porosity, high thermal and chemical stability, and convenient functionalization that promotes great potential in heterogeneous catalysis. In this article, we systematically present the structural design of CTFs as a versatile scaffold to develop heterogeneous catalysts for a variety of chemical reactions. We mainly focus on the functionalization of CTFs, including their use for incorporating and stabilization of nanoparticles and immobilization of molecular complexes onto the frameworks.

## 1. Introduction to Covalent Triazine Frameworks (CTFs)

**Scheme 1:**
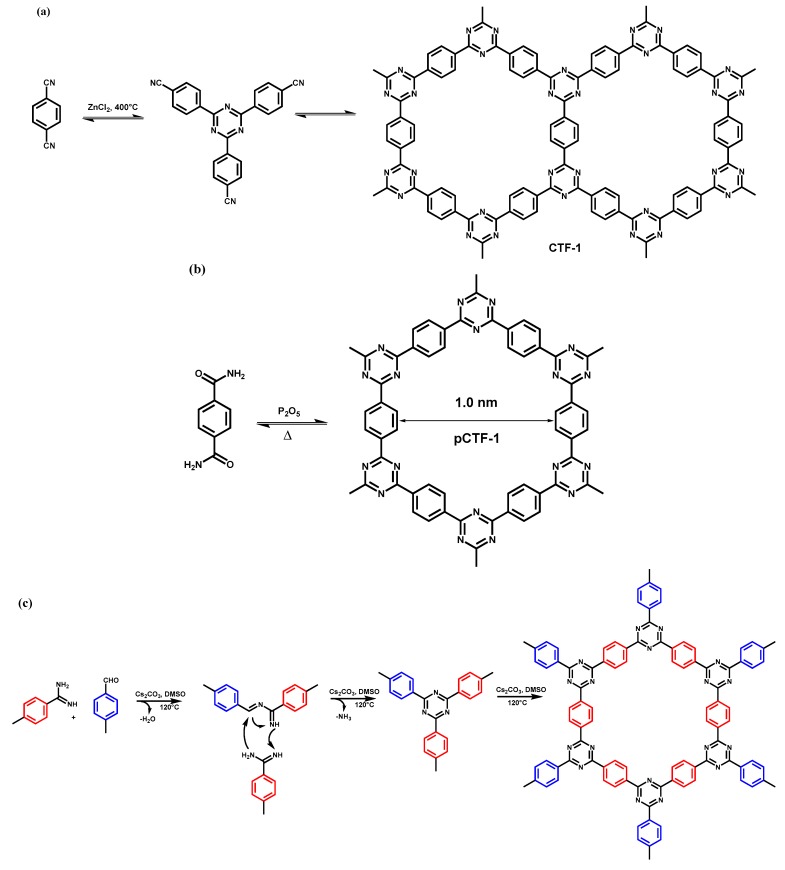
(**a**) Trimerization of 1,4-dicyanobenzene catalyzed by ZnCl_2_ under ionothermal reaction conditions for the formation of covalent triazine framework (CTF)-1; (**b**) Schematic representation of the P_2_O_5_-catalyzed direct condensation of terephthamide (TA) to a discrete *p*CTF-1; (**c**) Scheme showing reaction mechanism for CTF-HUST synthesis. (Modified and reproduced with permission from [4,15,16] respectively. Copyright 2008, 2018 and 2017, Wiley-VCH).

Covalent triazine frameworks (CTFs) represent a class of highly stable porous organic materials based on triazine linkages. CTFs are generally prepared through a simple trimerization of aromatic nitriles in the presence of a catalyst. The formation of triazine rings from nitrile compounds catalyzed by molten metal salts has been applied since the early 1960s [1]. G.H Miller (Texaco Inc.) extended this approach by introducing the potential of metal chloride as a good catalyst for the trimerization of a variety of aromatic nitriles [2]. The formation of thermally stable, insoluble and infusible polymeric materials occurs at elevated temperatures of 410 to 550 °C. These triazine containing polymers possess rich electron-deficient structures and have gained attention in supramolecular chemistry because of their strong tendency for hydrogen bonding, metal chelation and π-π interactions [3]. In 2008, Thomas and co-workers adapted the concept and introduced the synthesis of CTFs catalyzed by ZnCl_2_ under ionothermal reaction conditions which produces permanent porosity and crystallinity. They first reported CTF-1 with a surface area of 790 m^2^·g^−1^ and a pore volume of 0.40 cm^3^·g^−1^ which was obtained from the trimerization of 1,4-dicyanobenzene in molten ZnCl_2_ in a closed quartz ampule at 400 °C for 40 h (Scheme 1a) [4].

Structurally, most of the reported CTFs are amorphous because of the harsh synthesis conditions and high stability of the triazine linkage, yet they exhibit high specific surface area and permanent porosities [5]. In addition, the chemical structures and functionalities of this porous material can be adjusted by varying the structure of the monomer used as the building block. The introduction of heteroatoms or functional groups into the frameworks enhance the potential of CTFs for various applications especially gas storage, adsorption and catalysis.

The utilization of CTFs for various applications have been reviewed in several reports. Jin et al. reported the synthesis and applications of CTFs for energy and environmental purposes [6]. Tan et al. reviewed the development and challenges in the synthesis and potential applications of CTFs in gas adsorption and separation, energy storage and conversion, photocatalysis as well as heterogeneous catalysis [7]. In addition, our group has reported the functionalization of CTFs with nitrogen rich functionalities, (pyridine-4,2,6-triyl)-based CTF, and fluorine-containing hydrophobic CTF. The synthesized materials effectively enhanced the adsorption properties and selectivity towards CO_2_ and H_2_ due to stronger interaction between the gas molecules and the functionalized CTF materials [8,9]. Furthermore, we also demonstrated the potential of CTFs as a platform for the encapsulation of γ-Fe_2_O_3_ for implementing it as an excellent and novel adsorbent toward remediation of inorganic contaminants in water [10].

The presence of stoichiometric and well-defined triazine moieties in the framework makes CTFs very attractive materials as scaffold for metals, especially in heterogeneous catalysis [11]. They are particularly interesting owing to their high basicity due to the nitrogen-containing triazine moieties, exceptional chemical inertness, especially in acidic and basic media, and outstanding stability over a broad range of temperatures and pressure. Furthermore, their high surface area and porosity with rigid pore structures enables the facile diffusion of substrates and products during catalysis.

The application of CTFs in catalysis is a rapidly developing interdisciplinary research field. A number of reviews have been reported toward the applications of triazine-based polymers in heterogeneous catalysis [12,13]. This paper will present an in-depth review on the recent advances in preparation and functionalization of CTF materials for heterogeneous catalysis, and focuses on the development of CTFs as catalyst supports with an emphasis on the application in synthetic organic chemistry.

## 2. Design and Synthesis Methods of CTFs

Over the last decade, several procedures for the synthesis of CTFs have been developed. Porous CTF materials having a desired topology and functionality can be constructed from various monomers with specific functional groups, under suitable reaction conditions. For instance, CTFs can be prepared through the trimerization of aromatic nitriles in the presence of acidic catalysts [4,14], polycondensation reaction of monomer containing specific functional groups [15,16], and cross-coupling reaction of triazine containing building units [17,18]. However, for catalytic applications, the surface area and pore size, as well as the stability during the catalytic reaction conditions and reusability are important criteria for the material assessment. Especially for catalytic purposes, the accessibility of immobilized metal species, specific surface area and porosity play a major role especially in terms of mass transfer throughout the catalyst and eventually the overall catalytic activity. The following sections contain various methods used to synthesize CTFs and their effect on the end-product.

### 2.1. Trimerization of Aromatic Nitriles

Different conditions can be used for the trimerization of nitrile compounds leading to a triazine unit. The most common trimerization of nitriles is conducted under ionothermal conditions in molten ZnCl_2_ at 400 °C for 40–48 h in a closed quartz ampule [4]. Molten ZnCl_2_ is a good catalyst for the trimerization reaction and has a highly soluble reaction medium for the nitrile compounds. Harsh reaction conditions are applied to ensure the reversibility of the cyclotrimerization reaction [19]. However, the high stability of the triazine linkage causes low reversibility of the polymerization process. Hence, most of the obtained CTFs have a low crystallinity or show a limited long-range order to which results into the formation of amorphous structures. To date, only CTF-0, CTF-1 and CTF-2 have been successfully synthesized as crystalline CTFs using the ZnCl_2_ route [4,20,21]. An excess amount of ZnCl_2_ as the molten solvent as well as an increase in the synthesis temperature, results in an increased specific surface area and porosity of CTFs. However, partial decomposition occurs at elevated temperatures causing a loss of nitrogen and the creation of structural defects and thus results in mainly amorphous materials. Besides, synthesis at a temperature below 350 °C using extended reaction times up to 168 h leads to the formation of oligomeric products without any porosity [22].

An advanced synthetic protocol involves the addition of a salt template during the polymerization of CTFs to introduced mesopores into a microporous material without the loss of nitrogen content in the framework. In this approach, binary mixtures of ZnCl_2_ with various alkali chlorides such as LiCl, NaCl, and KCl were used as a reaction medium for the trimerization of nitriles [23]. The synthesis was performed at 300 °C during 60 h to initiate the formation of oligomers and subsequently, the samples were heated to 450 °C for an additional 5 h for complete polymerization. The salt-templated materials exhibit a significantly increased specific surface area and lead to an enormous increase of the mesopore volume up to ten times compared to the conventional method for CTF-1. However, the solubility of the used organic species in the corresponding salt melt turned out to be crucial for the generation of porous CTFs.

Another approach to reduce the polymerization reaction time is by using microwave (MW)-assisted ionothermal synthesis [24]. ZnCl_2_ is a good MW absorber, and high porosity CTFs were obtained easily within 10–60 min. However, the fast heating rate and high reaction temperature led to carbonization and rather low nitrogen contents.

The trimerization of aromatic nitriles can also be catalyzed by CF_3_SO_3_H in CHCl_3_ at room temperature or even within a microwave-assisted condition [14]. Despite mild reaction conditions that avoid the incipient carbonization of the materials, this approach cannot be applied to all functional nitriles building blocks and also the CTFs produced using this method exhibited low to moderate Brunauer–Emmett–Teller (BET) surface area compared to the ionothermal synthesis route. Hence, the ZnCl_2_ catalyzed trimerization of aromatic nitriles is the most practical approach due to the ease of synthesis and feasibility for a variety of functional building units, which in turn can be utilized for heterogeneous catalytic applications. However, the presence of residual Zn in the CTF even after a thorough cleaning cannot be ignored. Usually, at the end of the synthesis, the CTF is formed as a monolith which is crushed into a powder before carrying out the cleaning treatments. Firstly, the CTF is stirred in diluted hydrochloric acid and water to remove the residual Zn and Cl ions. Hereafter, washing steps in organic solvents such as tetrahydrofuran or acetone are used to remove the unreacted moieties from the material. In most of the reported papers, these washing steps are used directly, or a slightly modified procedure is followed. Nevertheless, it is important to note that a complete removal of ZnCl_2_ remains a challenge.

### 2.2. Polycondensation Synthesis Route

The triazine-based frameworks can also be synthesized through direct condensation of aromatic amides catalyzed by phosphorus pentoxide (P_2_O_5_) (Scheme 1b) [16]. The polymerization of amides catalyzed by P_2_O_5_ was conducted in a degassed flame-sealed ampule at a temperature up to 400 °C. The resulting CTFs exhibit not only a high specific surface area and stability but also a high crystallinity. Because of the lower sublimation temperature of the amide monomers, an appropriate temperature for the synthesis of CTFs is crucial in order to ensure better structural properties of the frameworks.

Another approach is by a polycondensation reaction of aldehydes and amidines in DMSO at 120 °C in the presence of cesium carbonate (Cs_2_CO_3_) as a base (Scheme 1c) [15]. The condensation reaction involves a Schiff base formation followed by a Michael addition. Furthermore, this one-pot polymerization of CTFs can be scaled up to multigram level because of the relatively low synthesis temperature. An advanced condensation approach can be utilized for the formation of crystalline CTFs by in situ formations of the aldehyde monomers through the controlled oxidation of alcohols [25]. This occurs by the slow oxidation of the alcohol into the aldehyde monomer in the dimethyl sulfoxide (DMSO) solution, and the reaction temperatures were subsequently increased to boiling point. The reaction temperature plays a critical role in this polycondensation reaction. By performing the polymerization reaction at a high temperature, the alcohol will oxidize rapidly to form an aldehyde and generates a high concentration of nuclei to form small particles, and thus only soluble oligomers or fragments are formed, while polymerization reaction at a temperature below boiling point mainly yielded a low degree of crystallinity of CTFs. Therefore, it is very crucial to keep the polymerization reaction at a lower temperature in the initial stage to control the in-situ formation of aldehyde and then maintain a higher temperature to boiling point to enhance the polymerization rate to improve the crystallization of the CTFs.

### 2.3. Cross-Coupling of Triazine-based Building Blocks

**Scheme 2:**
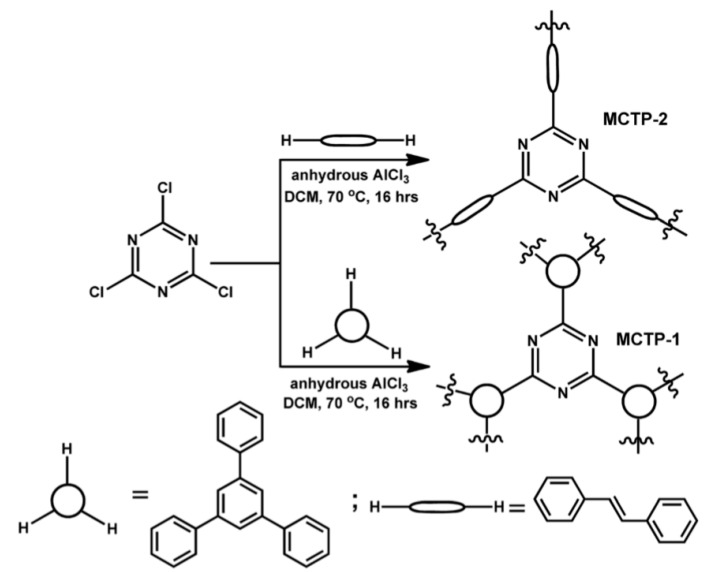
Friedel-Crafts synthesis for microporous covalent triazine polymer formation. Reproduced with permission from [17], Copyright 2015, Royal Society of Chemistry.

Microporous triazine-based polymers can also be derived from triazine containing building blocks through a relatively simple cross-coupling reaction such as Friedel-Crafts and Yamamoto homo-coupling. In the Friedel-Crafts reaction, cyanuric chloride acts as the triazine monomer which reacts with aromatic compounds in the presence of AlCl_3_ as Lewis acid catalyst (Scheme 2) [17,26,27]. Generally, the cross-coupling reaction is performed under reflux in dichloromethane (DCM) and is feasible for various aromatic components. This method produces microporous CTFs with a specific surface area of up to 1668 m^2^·g^−1^, possessing a low crystallinity due to the irreversible polymerization linkage. Also, this synthetic approach is unfavorable from an of economic and ecological perspective and is not a scalable reaction.

In a similar manner, CTFs can also be derived by a Ni-catalyzed Yamamoto reaction between 2,4,6-tris-(4-bromo-phenyl)-(1,3,5-triazine) and Ni(cod)_2_ catalyst in the presence of 2,2′-bipyridyl in dehydrated dimethylformamide (DMF) under inert conditions at 105 °C [18]. Porous CTFs with a specific surface area of up to 2015 m^2^·g^−1^ and a pore volume of 1.36 cm^3^·g^−1^ were obtained.

Though several synthesis methods have been developed for the preparation of CTFs, it is very hard to make a decision on which of these methods is the preferred option. The ionothermal synthesis route uses harsh conditions which result in materials that are mainly amorphous due to partial carbonization and side reactions. This makes the characterization of the material cumbersome. However, as this is a straightforward method it has been the most applied method for the synthesis of CTFs for several applications such as catalysis and gas adsorption. Particularly in catalysis, these CTFs have been used as highly stable supports, providing a high recyclability with limited loss of activity. However, it is important to note here that it is difficult to implement the ionothermally synthesized CTFs in photocatalysis. Due to carbonization, the end- product is a black colored powder which is mostly not photoactive. For this purpose, CTFs synthesized through more milder routes (Bronsted acid, amidine method and in situ oxidation) are better. The Bronsted acid assisted method has however a few restrictions, as it does not produce crystalline materials and it is not compatible with all functional groups. Particularly for monomers containing heteroatoms, this method will often not work. Hence for photocatalysis, the amidine method and in situ oxidation method are the preferred options. The in-situ oxidation method provides additional benefits as materials with a higher crystallinity are obtained which is essential for an efficient charge transfer in photocatalysis. Therefore, in conclusion, the choice of the ideal synthetic route to produce a CTF depends to a large extent on the envisioned application.

### 2.4. Characterization of CTFs

Underneath an overview is given of the most common and essential methods required to characterize CTFs. However, it is important to note that the presented list is not limited to only these methods but also other methods such as Raman spectroscopy, atomic force microscopy (AFM) etc. have been used in literature. Firstly, powder X-Ray diffraction (PXRD) is currently the most frequently used characterization method to confirm the structure of covalent organic frameworks (COFs). However, in the case of CTFs, this is difficult since most of the CTFs are obtained as amorphous powders. Nevertheless, in some reports CTFs have been obtained as crystalline powders from which a crystal model could be built [4,20,21]. Since the structure of the CTFs can be predicted based on the selected monomers, an initial crystal model can be built easily. This model can be optimized in a following step in correspondence to the best experimental PXRD pattern. A second essential characterization method for CTFs is Fourier-transform infrared (FT-IR) spectroscopy. FT-IR allows to determine the characteristic stretching and vibrating modes of the different functional groups present in the applied monomer during the CTF synthesis. Apart from the functional groups present in the monomer, the triazine group has characteristic bands around ~1510 and 1350 cm^−1^ that need to be verified in the end-product. In the ionothermally obtained CTFs, it is important that the -CN stretching peak at 2260–2222 cm^−1^ is not present anymore in the final product as this confirms the complete conversion of nitrile into triazine. Apart from FT-IR, elemental analysis provides essential information on the overall elemental content in the material. An ideal crystal model is required to determine the unit cell for comparing the theoretical elemental content to the experimental values. However, for most of the CTF structures, the crystal model is not available due to the absence of resolvable PXRD patterns. Nevertheless, researchers use in many cases, the predicted ideal model to calculate the theoretical elemental content to compare it to the experimentally obtained values. An important value to calculate is the C/N ratio. A comparison of this value with the theoretical C/N ratio allows to determine the degree of carbonization that occurred during the synthesis. This also allows to estimate the loss of nitrogen in the CTFs. Solid-state nuclear magnetic resonance (ss-NMR) spectroscopy has been regularly used to characterize insoluble materials. In case of CTFs, ss-NMR can provide details on the chemical composition and exhibit characteristic peaks for the functional groups present within the material.

In addition, nitrogen/Argon sorption measurements are the most important technique to characterize the porous properties of any material. The surface area, pore size distribution and pore volume can be obtained by using this method. It also may provide details on the micro/meso-porosity of the material. Especially during catalysis, porosity is important to allow a fast substrate diffusion and for the anchoring of homogeneous metal complexes or nanoparticles. After the anchoring step, this method can be applied to give an indirect indication on the success of the anchoring and the guest-framework interactions. Transmission electron microscopy (TEM) and scanning electron microscopy (SEM) are also commonly used techniques to determine the morphology of the CTFs. TEM provides details on the inner surface of the material, whereas SEM can give information on the surface composition. Often TEM and SEM combined with energy dispersive X-ray (EDX) spectroscopy is used to obtain details on the elemental composition. X-ray photoelectron spectroscopy (XPS) is also used to determine the elemental composition along with the bonding nature of the elements present in the CTF. It is, however, important to note that one must be careful in using the elemental composition obtained from XPS as it is only a surface technique and does not provide information on the bulk material itself. Thermogravimetric analysis (TGA) provides information on the stability of the material at elevated temperatures. In addition, the stability of CTFs is often examined under strong acidic, basic and solvent treatments. The PXRD or porosity measurements before and after the treatment are a good indicator for the stability of the materials.

## 3. CTFs as Support for Heterogeneous Catalysis

CTFs have emerged as a novel platform for high-performance heterogeneous catalysis owing to their rigid structures, thermal stability, as well as their high acid-base resistivity in comparison to other porous organic frameworks. The most significant advantages of CTFs are the well-defined nitrogen species in their triazine units and the ease of functionalization of the organic ligand in order to post-modify the CTF with active metal species to obtain heterogeneous catalysts. Furthermore, their high specific surface area and porosity provide a good accessibility of the active sites, and heteroatoms contribute to the stabilization of the active species. The following sections are divided into two—metal nanoparticle and molecular metal-based catalysis. Table 1 summarizes various CTFs used for different catalytic reactions. Though CTFs have been utilized for several types of catalysis (electrocatalysis, photocatalysis, organocatalysis), we have focused this review towards liquid phase organic transformations.

### 3.1. Support for Metal Nanoparticles

N-heterocyclic moieties in the CTFs are beneficial for improving the stability of nanoparticles, especially for liquid phase catalytic reactions. Also, the porous surface of the support can offer steric restriction to prevent the growth of metal clusters which causes agglomeration and thus deactivation of the active catalyst species.

**Scheme 3:**
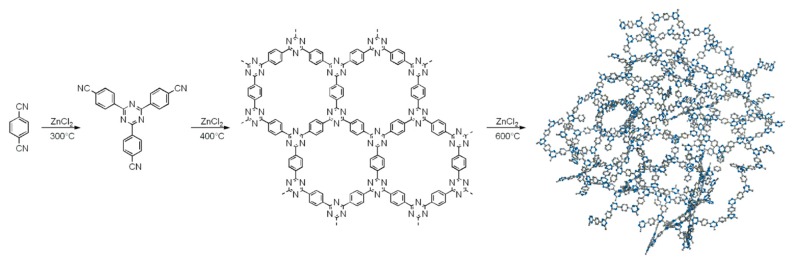
Reaction scheme for the dynamic trimerization of 1,4-dicyanobenzene toward the formation of CTF (Adapted with permission from [28]. Copyright 2010, American Chemical Society).

Thomas et al. immobilized Pd nanoparticles (Pd NPs) onto the CTFs materials to develop an active and efficient catalyst for the oxidation of glycerol in a liquid phase reaction system [28]. The CTF support was synthesized through the trimerization of 1,4-dicyanobenzene using ZnCl_2_ at 400 °C and then heated up to 600 °C for local expansion of the network. Surface areas up to 2814 m^2^·g^−1^ and pore volume of 1.79 cm^3^·g^−1^ were obtained (Scheme 3). Further on, the Pd NPs-supported CTF (Pd_PVA_/CTF) were prepared by a sol immobilization technique (NaBH_4_/polyvinyl alcohol (PVA)) to ensure better dispersion of the small NPs on the solid support. Pd_PVA_/CTF exhibited a higher activity and selectivity than Pd-supported activated carbon (Pd/AC) toward the formation of glycerate, and showed a high stability up to three cycles. Furthermore, Pd_PVA_/CTF was more resistant to deactivation which was attributed to the stabilizing effect achieved through the nitrogen functionalities present in the CTF materials.

**Figure 1:**
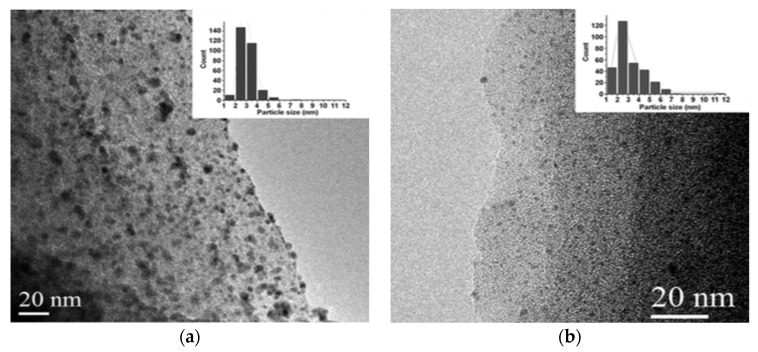
TEM images of Pd_PVA_/CTF (**a**) and Pd_IMP_/CTF (**b**). Insets: Particle size distributions of the Pd NPs (Reproduced with permission from [29]. Copyright 2015, Wiley-VCH).

The applied preparation technique of immobilizing Pd NPs onto the CTFs was observed to influence the activity and stability of the Pd/CTF catalyst in this catalytic reaction. The preparation of Pd_IMP_/CTF through the impregnation method led to the confinement of the Pd particles within the pores of the CTF (Figure 1) [29]. A slight decrease was observed in the activity and selectivity for the Pd_IMP_/CTF compared to the Pd_PVA_/CTF which could be attributed to the lower Pd exposure in the Pd_IMP_/CTF sample. Remarkably, owing to the physical confinement of the Pd NPs within the CTF materials, Pd_IMP_/CTF showed superior durability up to nine cycles.

**Figure 2:**
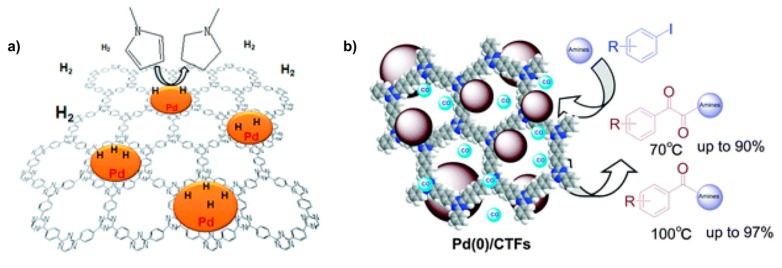
Schematic picture of (**a**) Pd/CTF catalyzed hydrogenation of *N*-heterocycles (Reproduced with permission from [32]. Copyright 2015, Royal Society of Chemistry); (**b**)Pd/CTF catalyzed carbonylation of aryl iodides with amines (Reproduced with permission from [33]. Copyright 2016, Royal Society of Chemistry).

Prati et al. examined the influence of the N-containing support on the catalytic activity and stability of the Pd system in benzyl alcohol oxidation [30,31]. The Pd NPs-supported CTF (Pd/CTF), activated carbon (Pd/AC), carbon nanotubes (Pd/CNTs) and nitrogen-doped carbon nanotubes (Pd/N-CNTs) were prepared by the sol immobilization method. Indeed, the triazine-rich CTF material with the highest nitrogen content significantly increased the Pd-catalyst performance compared to AC- and CNT-based support, which can be ascribed to the presence of N-functionalities which limit the growth of the NPs and contributes to a positive effect toward particle dispersion avoiding aggregation of NPs. Moreover, He et al. highlighted the enhanced catalytic activity of Pd/CTF in the hydrogenation of *N*-heterocyclic compounds compared to the traditional Pd/AC (Figure 2a) [32]. They also indicated that the well dispersed and uniform distribution of Pd NPs onto the CTF and excellent catalytic activity were attributed to the electron-donation from the N-moieties in the CTF to the Pd NPs which exhibited strong electronic metal-support interaction. Zhang et al. also reported the high activity and selectivity of Pd/CTF for the carbonylation of aryl iodides with amines (Figure 2b) [33]. The Pd/CTFs were prepared by the impregnation-reduction method (PdCl_2_/NaBH_4_) and they catalyzed the synthesis of α-ketoamides under an atmospherical pressure of CO without any specific additives, for which the typical reaction of Pd-catalyzed double carbonylation of aryl halides required additives such as amines and high pressures of CO to drive the catalytic reaction.

**Scheme 4:**
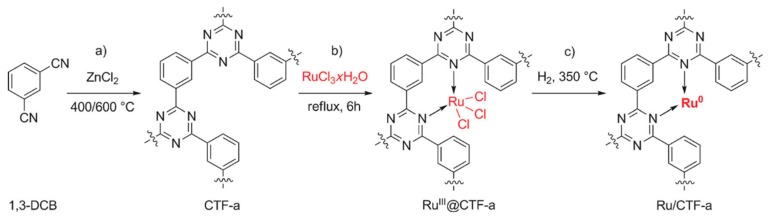
Schematic representation of the coordination and stabilization of Ru NPs onto CTF material (Reproduced with permission from [34]. Copyright 2015, Wiley-VCH).

Palkovits et al. developed Ru/CTF catalysts for the selective oxidation of 5-hydroxymethylfurfural (HMF) [34,35]. Ru NPs were distributed onto various CTFs using the coordination-reduction approach in which the RuCl_3_·xH_2_O precursor in ethanol was refluxed in the presence of the CTF materials, and the resulting coordinated Ru-species were reduced under H_2_ atmosphere (Scheme 4). The reported study showed that the enhanced catalytic performance of Ru-supported CTF not only depended on the surface area and porosity of the prepared materials but also was based on the N-content and the inherent polarity of the catalyst. Furthermore, the CTF-based support catalysts were far more active compared to the conventional Ru/C, and Ru/Al_2_O_3_ catalysts and additionally, the N-functionalities provided stabilizing effect for the Ru species, enabling an easy recycling of the catalysts. Ru/CTFs were also utilized as a selective catalyst for the conversion of xylitol to glycols with yields up to 80% [36]. The catalytic results revealed that the increased N-content in CTF-supported Ru catalysts suppressed the decarbonylation reactions and hence resulted into a high selectivity toward the desired products.

**Scheme 5:**
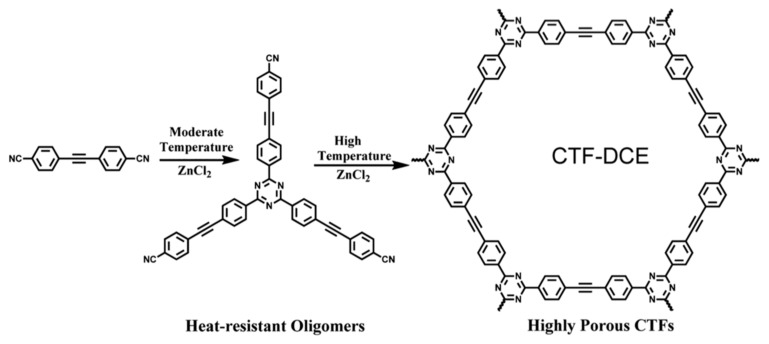
Polymerization of di-(4-cyanophenyl)ethyne (DCE) under ionothermal synthesis conditions (Reproduced with permission from [37]. Copyright 2018, American Chemical Society).

Zhang et al. synthesized a novel porous CTF-based on di-(4-cyanophenyl)ethyne building units (CTF-DCE) to anchor Ag species for the terminal alkyne carboxylation reactions under atmospherical pressure [37]. The CTF-DCE was synthesized under the ionothermal synthesis strategy through a sequence of the heat-resistant oligomer formation at moderate temperature to a highly porous CTF at high temperature with a BET surface area of 1355 m^2^·g^−1^ and pore volume of 0.93 cm^3^·g^−1^ (Scheme 5). The CTF-DCE supported-silver (CTF-DCE-Ag) was prepared by treating the CTF material with AgNO_3_ in hot DMSO at 80 °C. FTIR, PXRD and XPS analysis confirmed the deposition of metallic Ag^0^ onto the triazine moiety of the CTF-DCE. The CTF-DCE-Ag exhibited a six-fold higher TON compared to the Ag@MIL-101 catalyst for CO_2_ capture and conversion into propiolic acids under similar catalytic reaction conditions. The CTF-DCE-Ag could be recovered and reused up to five runs without any significant decrease in activity and, thus indicated the stability and recyclability of the catalyst.

### 3.2. Immobilization of Molecular Metal Catalysts

CTFs have been targeted as catalyst support for molecular complexes because of their exceptional chemical inertness toward acidic and basic media, combined with their outstanding stability over a broad range of temperatures and pressures enable CTFs to be particularly interesting for sustainable development in heterogeneous catalysis. To date, the aim to design CTF materials with strong electron-donating based ligands to provide high efficiency, good selectivity, and robust stability have been targeted by several research groups. The design of organic functionalized ligand-based CTF supported transition metal complexes for specific catalytic applications are summarized below.

**Scheme 6:**
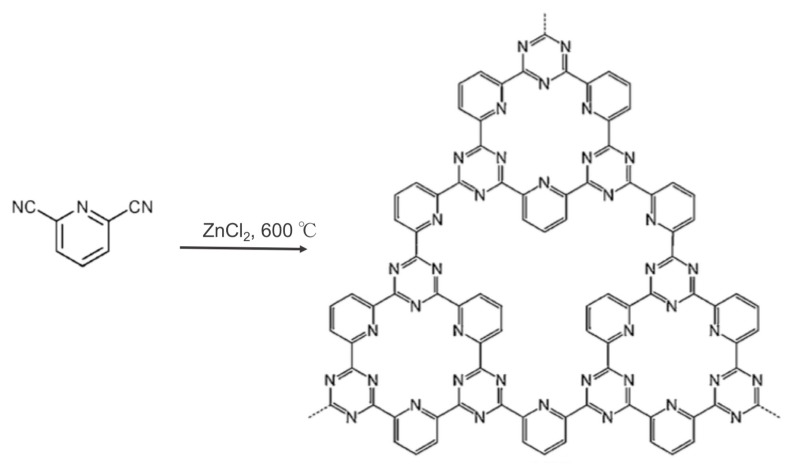
Schematic representation of the synthesis of the pyridine-based CTF [19].

Nitrogen-based ligands such as pyridine and bipyridine have been widely applied as homogeneous active transition metal catalyst supports. Functionalization of strong binding sites to a framework leads to various catalytic applications through efficient and stable heterogeneous based catalysts. Nitrogen-rich microporous CTFs derived from pyridine containing building units have been reported by Thomas et al. These materials were synthesized by a trimerization reaction under ionothermal conditions at temperatures up to 600 °C (Scheme 6) [19]. The resulting pyridine-based CTF with a specific surface area of 1061 m^2^·g^−1^ and a pore volume of 0.93 cm^3^·g^−1^, contained numerous bipyridyl moieties that are accessible and enable anchoring of a transition-metal complex.

Schüth et al. reported the potential of pyridine-based CTF material as a solid matrix for Pt(II) complex catalyzed methane oxidation [38]. The platinum species were introduced onto the CTF using two different routes; by an in-situ reaction of CTF and the Pt(II) precursor in the catalytic reaction mixture (K_2_[PtCl_4_]-CTF), or by pre-coordination of the Pt(II) complex onto the CTF (Pt-CTF). Both Pt-modified CTF catalysts (K_2_[PtCl_4_]-CTF and Pt-CTF) exhibited a high activity and selectivity for the oxidation of methane to methanol under extremely harsh reaction conditions at 215 °C in concentrated sulfuric acid (30% SO_3_) and high pressure of CH_4_ (40 bar). The Pt-CTF catalyst showed a high stability over several runs with a TON above 250. The K_2_(PtCl_4_)-CTF catalyst showed a deactivation after the fifth run. The platinum sites were analyzed by XPS before and after catalysis, revealing the Pt(II) to be the predominant species. It was observed that the K_2_(PtCl_4_)-CTF material possesses a lower amount of incorporated Pt species than Pt-CTF.

**Figure 3:**
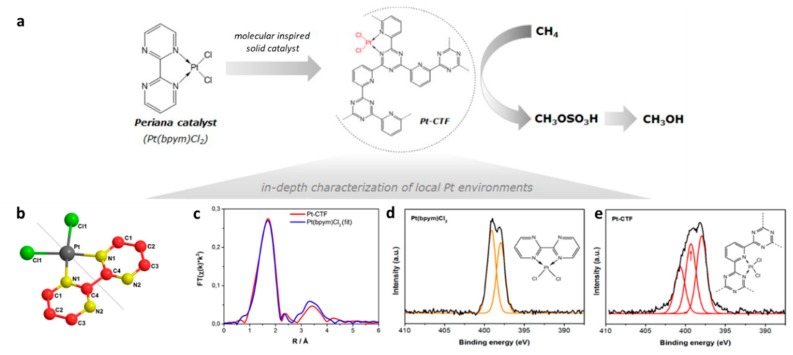
(**a**) Schematic diagram of the molecular Pt-CTF catalyst having a similar coordination environment as the molecular Periana catalyst (Pt(bpym)Cl_2_) for the oxidation of methane to methanol in concentrated sulfuric acid; (**b**) Structural model of Pt(bpym)Cl_2_; (**c**) EXAFS analysis of Pt-CTF and the fitted theoretical Pt(bpym)Cl_2_ model; (**d**) XPS N 1s spectra of Pt-modified 2,2′-bypyrimidine ligand and (**e**) XPS N 1s spectra of Pt-modified CTF (Adapted with permission from [39]. Copyright 2016, American Chemical Society).

In a follow-up study, the local environments of the Pt sites within the Pt-CTF catalyst were identified to be an analogue of the molecular Periana catalyst, Pt(bpym)Cl_2_ (Figure 3) [39]. The analysis by EXAFS, XANES, XPS, and the surface characterization methods, provided evidence for the coordination of Pt(II) onto the pyridinic nitrogen sites in the Pt-CTF catalyst which are similar to one of the two coordination environments in crystalline Pt(bpym)Cl_2_. In addition, this study provides detailed insights into the underlying molecular compositions, structures, and distribution of a heterogenized Periana catalyst onto the CTF materials.

Gascon et al. reported the immobilization of an (IrCp*Cl_2_)_2_ complex on the pyridine based CTF material as a base free catalyst for the transfer hydrogenation reaction [40]. The Ir(III) complex coordinated to the bipyridyl moieties within the framework, resulted in an air- and moisture-stable Ir@CTF catalyst. XPS analysis revealed that the Ir species in the system are in the oxidation state 3+ and remained unchanged prior to and after catalysis. Under the optimized reaction conditions, the Ir@CTF (0.4 mol % Ir used in reaction) catalyzed the transfer hydrogenation of 1-octene-3-ol with a 98% conversion and 82% yield to the targeted 3-octanone, in the presence of isopropanol as solvent at 120 °C under an inert atmosphere (N_2_, 2 bar) for 23 h, without the need for additives. In a typical catalytic reaction of allylic alcohols, an additive such as bases or hydrogen acceptors are needed to promote the reaction. Most likely, the CTF material is not just an inert support for the Ir(III)Cp* complex dispersion, but rather the presence of the pyridine molecules plays an active role as co-catalyst by assisting the deprotonation and coordination of the initial alcohol onto the Ir center to form the initial metal enolate for the isomerization reaction. The turn-over frequency (TOF) of the Ir@CTF catalyst was 24 min^−1^, and outperformed other Ir(III)-based porous material catalysts. The recyclability of the Ir@CTF material was performed up to six consecutive runs. However, a slight decrease of activity was observed over the first three runs, while a more pronounced decrease of activity was observed afterwards. The catalyst deactivation was mainly attributed to build-up of adsorbed products on the catalyst surface, progressively blocking the active sites.

In another catalytic application of the pyridine-based CTF as catalytic support, the same group reported on the immobilization of a Ni(II) complex onto the CTF which catalyzed the oligomerization of ethylene [41]. Ni(II) was coordinated onto the CTF support by the impregnation of an excess of Nickel(II) bromide ethylene glycol dimethyl ether (DME*NiBr_2_) under mild conditions. The TEM micrographs revealed a good dispersion of Ni throughout the solid materials with no metallic nanoparticle formation and XPS analysis indicated the preferential coordination of Ni to the pyridinic N species within the CTF. The catalytic performance and active site accessibility influenced the selectivity of the catalyst in the ethylene oligomerization. The microporous Ni(II) supported pyridine-CTF catalyst showed higher selectivity to C_8_ than to C_6_. The slower diffusion of olefins throughout the pores of the materials causes a re-adsorption of the products, thus leading to further oligomerizations.

Palkovits et al. reported that the porosity and polarity of CTF supported Rh(III) catalysts significantly influence the catalytic activity in hydroformylation reaction [42]. Rhodium(III) chloride hydrate was immobilized onto various CTF materials having different porosities and surface polarities. Superior results were obtained with the Rh(III) catalyst supported onto the highly mesoporous and low polarity of 4,4′-dicyanobiphenyl based CTF, whilst the microporous and high surface polarity of the 2,6-dicyanopyridine based CTF support showed the lowest activity. This study demonstrates that the mesoporosity and accessibility of the active sites in combination with the polarity of the surface support offers high activity and selectivity for the solvent-free hydroformylation of 1-octene.

**Scheme 7:**
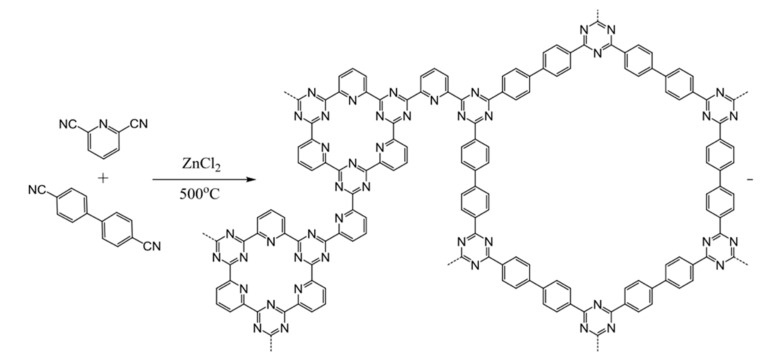
Synthesis of the mesoporous CTF from trimerization of 2,6-dicyanopyridine and 4,4′-dicyanobiphenyl under ionothermal synthesis conditions (adapted with permission from [43]. Copyright 2015, Wiley-VCH).

One of the great advantages of CTFs is that their solid-state properties can be adjusted by varying the starting building blocks. Gascon et al. designed a mesoporous CTF constructed of 2,6-dicyanopyridine and 4,4′-dicyanobiphenyl with the ratio of 1:2 (Scheme 7) [43]. The biphenyl monomer introduced the mesoporosity into the CTF while the presence of the pyridine sites in the framework not only provided an inherent basicity but also introduced bipyridine moieties that acted as metal support that assisted the activation of small molecules.

**Figure 4:**
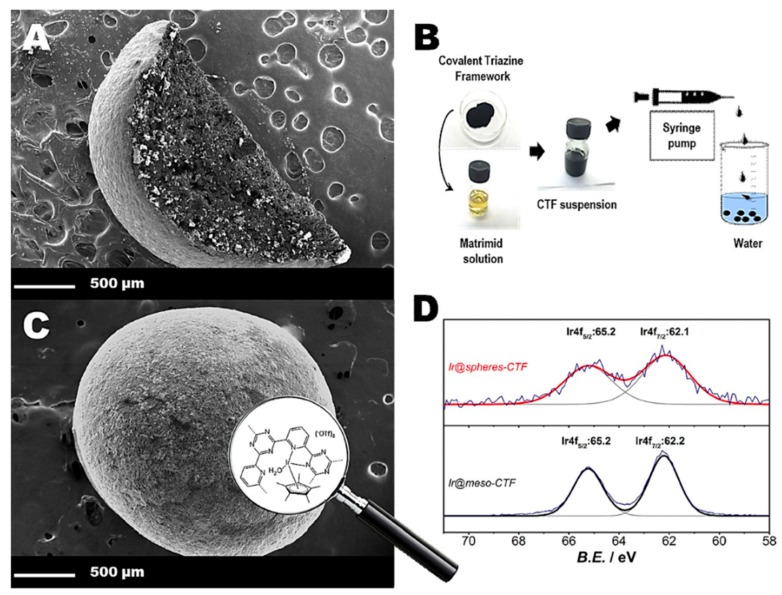
(**A**) SEM of a cut CTF-based sphere; (**B**) synthesis conditions for the shaping of CTF; (**C**) SEM image and its molecular structures of immobilized Ir complex on the CTF-based sphere; (**D**) XPS analysis of iridium species in the powder and shaped CTF catalysts (Reprinted with permission from [44]. Copyright 2016, Wiley-VCH).

To be more practical, in terms of catalyst recycling, the mesoporous CTF was shaped into a spherical form, without losing its properties. The CTF-based spheres were prepared by a phase inversion method using polyimide Matrimid^®^ as a binder (Figure 4) [44]. A phase inversion method refers to a process in which solvents are removed from a liquid-polymer mixture to produce a porous solid. In this case, the CTF-Matrimid suspension was loaded into a syringe and was pumped into water where the anti-solvent causes the phase inversion. The resulting CTF spheres showed a high thermal stability, porosity, and the possibility of coordinating metal clusters. In contrast to the powder-based materials, the spheres-based catalyst is more easily handled, and is fully recycled without loss of material through at least four consecutive runs. A highly efficient organometallic complex, Ir(III) Cp* was immobilized through coordination within the CTF spheres to render a molecular yet heterogeneous and stable catalyst, which is easy to handle and to recycle in the CO_2_ hydrogenation to formic acid. Moreover, utilization of shaped particles carries the advantage of facile catalyst recycling and improved reproducibility.

**Figure 5:**
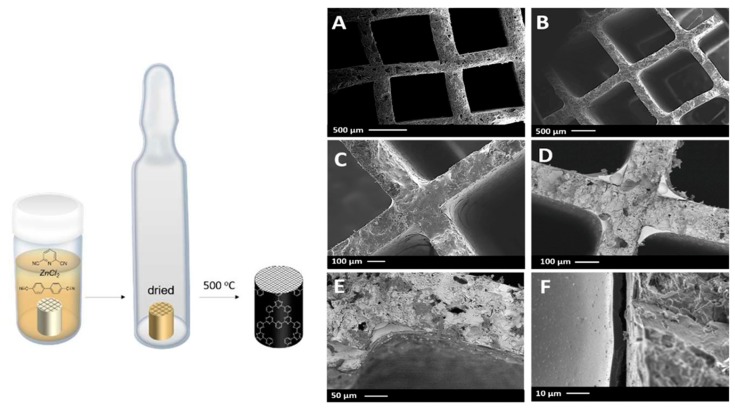
(**Left**) Representation of the coating procedure step. (**Right**) SEM micrograph of (**A**) CTF powders (**B–E**), CTF-coated monolith (**F**) view on the wall of a CTF-coated monolith (Adapted with permission from [45]. Copyright 2017, American Chemical Society).

However, the porosity of the CTFs could not be completely preserved upon formulation. To address this issue, and the diffusion limitations derived from heterogenization, Gascon and co-workers introduced a well-defined CTF coated on cordierite monoliths, prepared by quasi chemical vapor deposition (Figure 5) [45]. These coatings are stable, easy to handle, and can be used in the same way as the parent material to coordinate metal complexes. Extensive characterization demonstrates that these coatings contain similar properties as the powder-based materials. The monolith-supported CTF showed superior performance for the selective partial oxidation of methane to methanol, attributed to the better mass transport properties of the thin layer coated onto the monolith channels compared to the powder-form CTF.

**Scheme 8:**
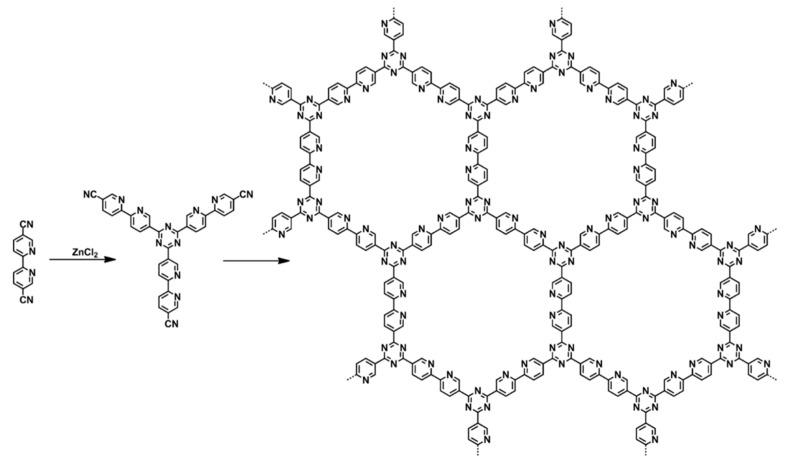
Schematic representation of the synthesis of the bipyridine-based CTF (bpyCTF) (Reproduced with permission from [46]. Copyright 2012, Royal Society of Chemistry).

Lotsch et al. developed a CTF based on a bipyridine-based building block with hierarchical microporosity and high specific surface areas of up to 1100 m^2^·g^−1^ in which the bipyridine units acts as scaffold to site-specifically coordinate the molecular metals including Co, Ni, Pt and Pd which are anchored on the CTFs by using wet impregnation method (Scheme 8) [46].

Yoon et al. utilized the bipyridine-based CTF (bpyCTF) as support for Ir- and Ru-complexes in the hydrogenation of CO_2_ to formate [47,48,49]. The XPS measurements revealed the complexation of both Ir and Ru ions onto the bpyCTF and showed that the coordination environment was similar to that of their homogeneous counterparts. Both catalysts converted CO_2_ into formate under mild reaction conditions in the presence of triethylamine as a base and demonstrated excellent recyclability over consecutive runs. The turnover number (TON) and turnover frequency (TOF) outperformed the conventional heterogeneous catalysts.

**Figure 6:**
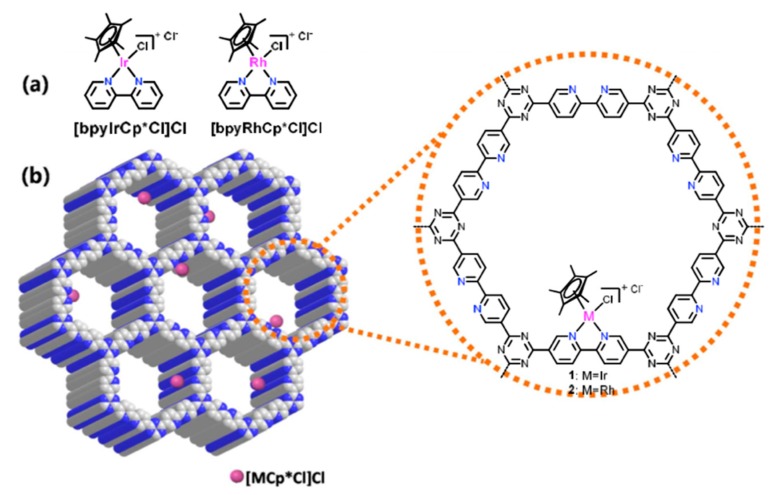
Structural representation of (**a**) homogeneous Ir- and Rh-based catalysts, and (**b**) CTF-supported catalysts (Adapted with permission from [50]. Copyright 2016, Royal Society of Chemistry).

Owing to the high stability of CTFs in acidic and basic media, Yoon et al. extended the study of the bpyCTF as catalytic support by incorporating the Ir and Rh half-sandwich complexes as active heterogeneous catalysts for the aqueous-phase hydrogenation of carbonyl compounds (Figure 6) [50]. Most of the MOFs and other transition metal-based oxide catalysts are unstable in acidic reaction conditions. Interestingly, these CTF supported heterogeneous catalysts (bpy-CTF-(Cp*IrCl))Cl and (bpy-CTF-(Cp*RhCl))Cl demonstrated to be efficient, recyclable, and have superior activities at acidic medium reaction (pH 3.5) for a broad substrate scope. In addition, these are also industrially viable catalysts for the exclusive production of alcohols in water.

The high specific surface area and good stability of the bpyCTF material in various catalytic conditions was further utilized by the group of Yoon et al. to immobilized a bimetallic (Al(OTf)_2_) (Co(CO)_4_) based catalyst onto the bpyCTF for the carbonylation of propylene oxide (PO) into β-butyrolactone [51]. The catalyst was prepared by the immobilization of Al(OTf)_3_ onto the bpyCTF, using a hydrothermal method. Subsequently, the OTf^-^ anions were exchanged with (Co(CO)_4_)^−^ anions. The nitrogen adsorption measurements proved that the presence of the anions did not completely block the pores of bpyCTF, to allow small molecules such as PO and CO to reach the active catalytic sites. XPS analysis revealed a similar coordination environment in the developed heterogeneous catalyst in comparison to the homogeneous catalyst. The active heterogeneous (bpy-CTF-Al(OTf)_2_)(Co(CO)_4_) catalyst converted PO into β-butyrolactone with a high selectivity of 90%, which is comparable to those of the homogeneous catalyst. Moreover, the catalyst was readily separated from the product and repeatedly used for several runs. The selectivity was maintained, although a slight reduction in the activity was also observed which may originate from partial discharge of the (Co(CO)_4_)^−^ anion from the catalyst upon product separation.

**Figure 7:**
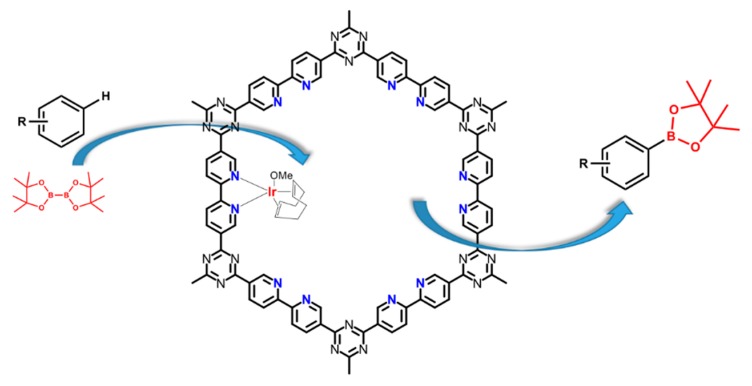
Structural representation of the Ir(I)@bpyCTF catalyzed CH boryation of aromatic compound in the presence of B_2_Pin_2_ as a boron source (reproduced with permission from [52]. Copyright 2019, Elsevier).

Very recently, our group reported the post-functionalization of bpyCTF with (Ir(OMe)(cod))_2_ complex towards the development of a highly stable and efficient catalyst for the CH borylation of aromatic compounds (Figure 7) [52]. The synthesized Ir(I)@bpyCTF catalyst exhibited excellent catalytic activity for CH borylation of arenes and heteroarenes in the presence of B_2_Pin_2_ as a boron source. Ir(I)@bpyCTF also exhibits high stability and reusability for at least five cycles without significant loss of activity. Physical characterizations combined with density functional theory (DFT) calculations elucidated the local geometry of the immobilized Ir(I)-complex onto the bipyridine-moieties in the framework. A similar coordination environment in comparison to its homogeneous counterpart was observed. More interestingly, Ir(I)@bpyCTF also showed similar catalytic trend as the homogeneous Ir-based catalysts and exhibited a better performance than the conventional Ir-based heterogeneous catalysts.

**Scheme 9:**
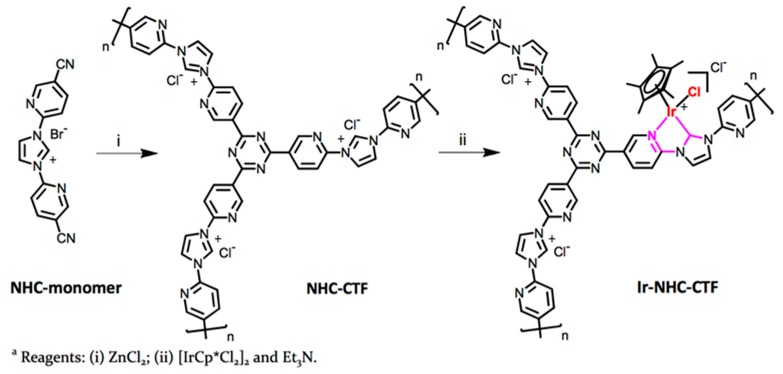
Schematic representation of synthesis of the Ir-NHC-CTF (Reproduced with permission from [53]. Copyright 2017, American Chemical Society).

The N-heterocyclic carbene (NHC) or imidazolium-based ligand possess strong σ-donating and poor π-accepting characters which can improve the electron density on the central metal ions that would enhance the efficiency of transition metal complexes heterogenized catalysts. Hence, Yoon and co-workers introduced the NHC-based CTF as catalyst support and employed it as an efficient heterogeneous catalyst for the CO_2_ hydrogenation to formate (Scheme 9) [53]. The Ir(III) complex was immobilized onto the NHC-functionalized CTF through N^C coordination site via a post-synthetic metalation strategy. Remarkably, the Ir-NHC-CTF system exhibited excellent activity with better TON and TOF in comparison to the values observed for the Ir-based bpyCTF and sphere-shaped Ir@CTF mentioned above for the same catalytic reactions. However, the activity of the catalyst decreased over three successive runs which is attributed to the Ir leaching during each cycle.

**Figure 8:**
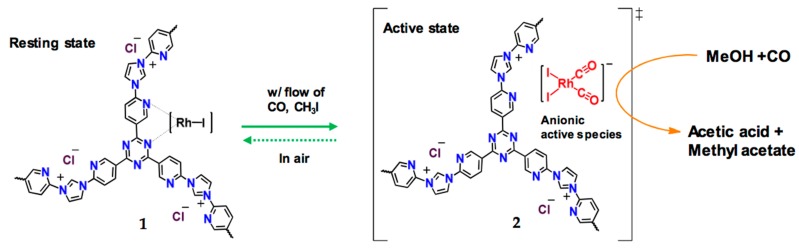
Structural representation of the Rh-bpim-CTF catalyst in resting state (**1**), and (**2**) the suggested structure of the in situ generated active state (Reprinted with permission from [54]. Copyright 2018, Royal Society of Chemistry).

The positive charged imidazolium-CTF was also employed as a support for Rh-based molecular catalysts in the carbonylation of methanol [54]. The characterization of the catalyst revealed that the Rh-complex was incorporated as a single-site catalyst throughout the support by ligation of the Rh complex to the abundant N-atom sites of the CTF (Figure 8). The Rh-bpim-CTF system showed a higher efficiency and long-term stability during the methanol carbonylation in plug-flow gas phase reaction compared to the Rh-bpy-CTF catalyst. The electron donation effect of the abundant N-atom sites in the CTF enhanced the nucleophilicity of the central metal ion and hence increased the catalytic activity. The strong ion-pair interaction between the Rh centers and the positive charged support dramatically increased the stability.

In another catalytic study, the same group of Yoon et al. reported on the potential of this charged imidazolium-based CTF to form complexes with (Co(CO)_4_)^−^ by maintaining the homonuclear ion pair and efficiently catalyzed the carbonylation of epoxide [55]. The imidazolium-CTF was treated with KCo(CO)_4_ in methanol under a CO atmosphere and subsequently a facile exchange of endogenous Cl^-^ anions with exogenous (Co(CO)_4_)^−^ anions occurred. XPS analysis showed that the coordination environment of Co anions present in the heterogenized catalyst was similar to that of the homogeneous system and further characterization by ICP-OES, SEM and elemental analysis collectively confirmed the existence of (Co(CO)_4_)^−^ in the frameworks. The heterogenized (imidazolium-CTF)(Co(CO)_4_) catalyst exhibited a much better selectivity in comparison to its homogeneous counterpart (Bmim)(Co(CO)_4_) catalyst for the production of methyl 3-hydroxybutyrate (MHB) from propylene oxide (PO) and CO. Typically, the formation of side products in this catalytic reaction are inevitable due to the nucleophilic attack of methanol on PO. Furthermore, the catalyst stability was revealed via the recycling experiments which shower that a small amount of (Co(CO)_4_)^−^ ions leached in every successive cycle.

**Figure 9:**
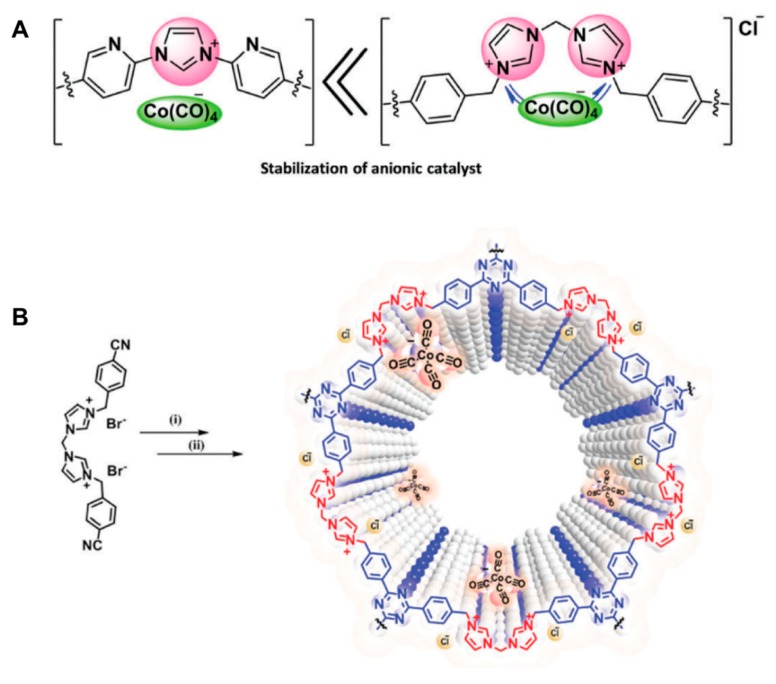
(**A**) Comparison of the intramolecular stabilization of anion in (bis-imidazolium-CTF-Cl)(Co(CO)_4_) (**right**) over (imidazolium-CTF)(Co(CO)_4_) complexes (**left**); (**B**) Representation of the synthesis of (bis-imidazolium-CTF-Cl)(Co(CO)_4_) (i) ZnCl_2_, 400 °C; 48 h (ii) KCo(CO)_4_, MeOH, 50 °C, 0.5 MPa of CO, 24 h (Adapted with permission from [56]. Copyright 2018, Royal Society of Chemistry).

The leaching of the metal active sites from the catalytic support resulted into a decrease in selectivity and recyclability of the catalyst. Thus, Yoon and co-workers designed a bis-imidazolium-based CTF as catalytic support to enhance the stability of the (Co(CO)_4_)^−^ ions during the catalytic process of the hydroesterification of epoxides (Figure 9) [56]. The catalytic results demonstrated that the bis-imidazolium-functionalized framework introduced intramolecular dication-anion interaction within the framework and literally activated more epoxide with better selectivity and decreased the (Co(CO)_4_)^−^ ions leaching from the CTF. This significantly increased the catalytic stability for epoxide carbonylation.

**Figure 10:**
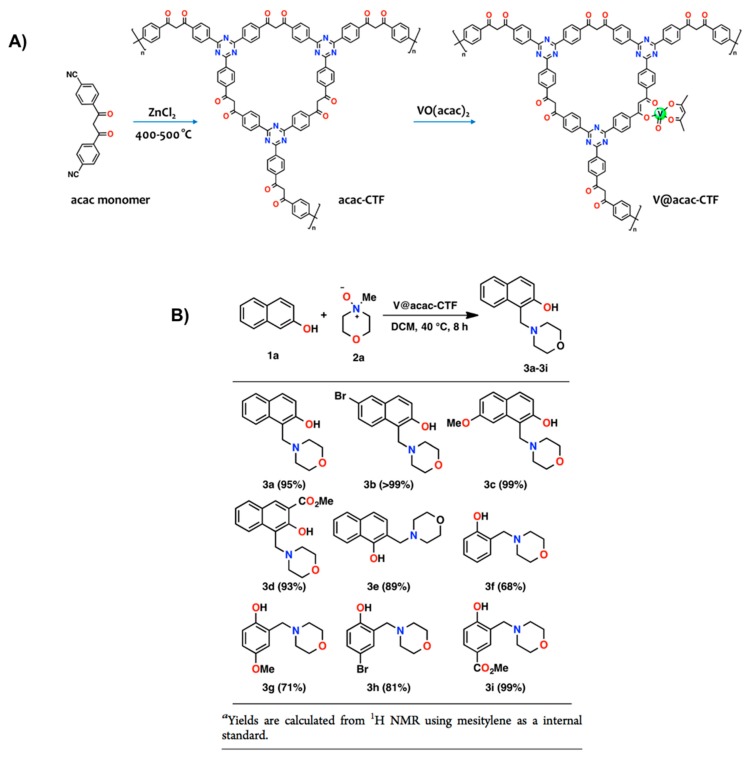
(**A**) Schematic representation of the synthesis of acac-CTF supported VO(acac)_2_ complex, and (**B**) V@acac-CTF catalyzed Mannich-type reactions with different substrates (Adapted with permission from [57]. Copyright 2018, American Chemical Society).

Acac-based transition metal complexes have shown significant catalytic performance in a wide variety of organic transformation such as cross-coupling reaction, alcohol oxidation, hydroxylation, etc. Our group designed CTFs functionalized with acetylacetonate (acac) groups (acac-CTF) to support a VO(acac)_2_ complex which efficiently catalyzed a Mannich-type reaction (Figure 10) [57]. The acac-CTFs were prepared from the trimerization of 4,4′-malonyldibenzonitrile under ionothermal synthesis conditions, and BET surface areas up to 1626 m^2^·g^−1^ were obtained. The acac-CTFs showed excellent CO_2_ and H_2_ storage capacity and good CO_2_/N_2_ selectivity. The V@acac-CTF catalyst was synthesized by a post-synthetic metalation of the acac-CTF material with VO(acac)_2_ in toluene. The strong metalation of the vanadium ions toward O^O coordination sites in the frameworks was confirmed by FTIR, ^13^C MAS NMR, and XPS analysis. The in-situ formation of iminium ions from tertiary amine oxides in the presence of V^4+^ anchored on acac-CTF facilitates the potential application in modified Mannich-type reactions. The V@acac-CTF showed outstanding reactivity and reusability with a TON of 213 for a wide substrates scope. The higher reactivity and reusability of the catalyst is attributed to the strong coordination of the vanadyl ions to the electron-donating based acetylacetonate groups in the framework.

## 4. Conclusions and Outlook

CTFs have attracted a great deal of attention especially in heterogeneous catalysis due to their inherent nitrogen groups and tunable functionalities that allow exquisite control over the chemical nature of the specific surface areas and physical properties of the resulting networks. They offer a number of advantages as porous solid catalysts towards selected challenges in the development and intensification of catalytic processes. CTFs can be utilized as a support for catalytically active metal nanoparticulate species wherein it can provide a stabilization effect compared to conventional porous solid supports. Additionally, the organic building units can be tailored synthetically with defined functional moieties to enable the immobilization of molecular metal complexes.

Despite the tremendous potential of CTFs as catalytic support, as demonstrated in this review, there are still challenges which need to be addressed in order to improve the application in organic synthesis and in industrial chemical production. Owing to their amorphous nature, their behavior in catalysis are often difficult to understand. The accessibility of active catalytic species in the framework is not always guaranteed, thus, the catalytic activity has always been determined by their TONs and TOFs calculated from the entire metal loading instead of accessible species. Although metal leaching is a limited issue, catalyst deactivation is still observed upon extended recycling tests. Further insight into the deactivation pathways is necessary for developing more robust catalysts with extended lifetime.

CTFs are becoming an exciting new type of porous organic support for heterogeneous catalysis owing to their well-defined nitrogen contents and high thermal and chemical stability. Although numerous catalytic reactions catalyzed by metal supported CTFs have been presented in this review, further development of CTFs as metal support are still highly desired, especially toward industrially relevant reactions, such as C–H functionalization and asymmetric catalysis.

## Figures and Tables

**Table 1:** Summary of various catalytic reactions performed by utilizing CTFs.

Material	Monomer	Metal content	Type of Reaction	Activity	Ref
Pd/CTF	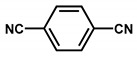	1 wt % Pd (nanoparticles)	Oxidation of glycerol into glyceric acid	Rate of glycerol mol·h^−1^·mol^−1^ consumption: 982 Selectivity = 81%	[28]
Pd/CTF	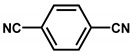	1 wt % Pd (nanoparticles)	Oxidation of Benzyl alcohol	Turn-over frequency (TOF) = 1453 h^−1^ Selectivity = 98%	[30]
Pd/CTF	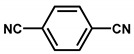	4 wt % Pd (nanoparticles)	Hydrogenation of *N*-heterocycles (1,10-phenanthroline)	Pressure/Selectivity = 30 bar/98.9%; 20 bar/97.9% for 8H-phen TOF = 47.6 h^−1^ (1st cycle)	[32]
Pd/CTF	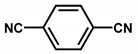	2.05 wt % Pd (nanoparticles)	Selective double carbonylation of aryl iodides	Several substrates tested Yield up to 90% with selectivity = 95%	[33]
Ru/CTF-c	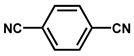	3.91 wt % Ru (nanoparticles)	Oxidation of 5-hydroxymethylfurfural to 2,5-furandicarboxylic acid	Conversion > 99% Yield = 41.4%	[35]
Ru/CTF-c	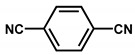	5 wt % Ru coordination using (RuCl_2_(p-cymene))_2_	Hydrogenolysis of xylitol	Full conversion with 15% selectivity to propylene glycol	[36]
CTF-DCE-Ag	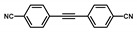	4.3 wt % Ag (nanoparticles)	Carboxylation of terminal alkynes	TON: 247 Yield > 98.9	[37]
Pt-CTF	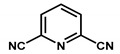	-	Low temperature oxidation of methane to methanol	Selectivity > 75% TON = 246	[38]
Ir@CTF	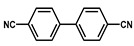	2.4 wt % Ir	Isomerization of 1-octen-3-ol to 3-octanone	TOF = 24 min^−1^	[40]
Rh@CTF-c	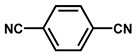	3.5 wt % Rh	Hydroformylation of crude 1-octene	62% conversion TOF = 600h^−1^ TON = 12,000	[42]
CTF-Ir	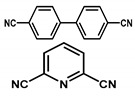	16 wt % Ir	Dehydrogenation of formic acid	TOF = 27,000 h^−1^ TON = 1,060,000	[43]
Ir@meso-CTF	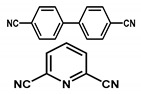	2 wt % Ir	Hydrogenation of CO_2_	TON = 358	[44]
Ir@meso-CTF@monolith	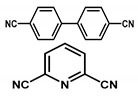	0.23 μmol/0.045 mg Ir	Dehydrogenation of formic acid	TOF = 207,200 h^−1^/TON = 2230	[45]
Bpy-CTF-(IrCp*Cl)Cl	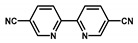	4.7 wt % Ir	Hydrogenation of CO_2_ to formate	TOF = 5300 h^−1^ TON = 5000	[47]
(bpy-CTF-Ru(acac)_2_)Cl	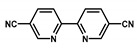	1.68 wt % Ru	Hydrogenation of CO_2_ to formate	TOF = 22,700 h^−1^ TON = 21,200	[48]
(bpy-CTF-RuCl_3_)	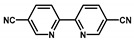	2.1 wt % Ru	Hydrogenation of CO_2_ to formate	TOF = 38,800 h^−1^ TON = 20,000	[49]
(bpy-CTF(RhCp*Cl)Cl) &(bpy-CTF(IrCp*Cl)Cl)	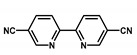	1.78 wt % Rh & 4.7 wt % Ir	Transfer hydrogenation of carbonyl compounds	Conversion = 99% Rh catalyst is more active than Ir catalyst. But, only Ir catalyst maintained activity during recycling.	[50]
(bpy-CTF-Al(OTf)_2_) (Co(CO)_4_)	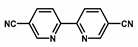	3.76 wt % Al & 2.67 wt % Co	Carbonylation of epoxides into β-lactones	Conversion > 99% Selectivity = 90%	[51]
Ir(I)@bipyCTF	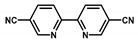	8.26 wt % Ir	C-H borylation of 1,2-dichlorobenzene	TON = 64 Yield = 95%	[52]
Ir0.68-NHC-CTF	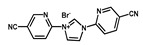	0.68 wt % Ir	Hydrogenation of CO_2_ to formate	TOF = 1600 h^−1^ TON = 24,300	[53]
Rh-bpim-CTF	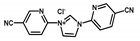	0.61 wt % Rh	Carbonylation of methanol	Conversion = 93% TOF = 2100 h^−1^	[54]
(imidazolium-CTF)(Co(CO)4)	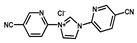	3.62 wt % Co	Direct Synthesis of Methyl 3-Hydroxybutyrate from Propylene Oxide	Conversion > 99% Selectivity = 86%	[55]
V@acacCTF	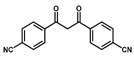	1.6 wt % V	Mannich reaction between 2-naphthol and *N*-methylmorpholine N-oxide	TON = 213 Conversion = 100% Yield = 95%	[57]

* For the annotation of the material, the annotation used in the literature was applied.
